# Lipopolysaccharide-mediated macrophage polarization, conserved pathogenesis, and implications for peripheral neuropathy: a systematic review

**DOI:** 10.1007/s00011-026-02317-3

**Published:** 2026-07-15

**Authors:** Leah Elson, Niels Eijkelkamp, J. Henk Coert

**Affiliations:** University Medical Center Utrecht, Room Number G04.126, P.O. Box 85060, 3508 AB Utrecht, Netherlands

**Keywords:** Gut-nerve axis, Peripheral neuropathy, Gut dysbiosis, Lipopolysaccharide

## Abstract

**Objective and design:**

This systematic review synthesized evidence for a conserved lipopolysaccharide (LPS)-mediated pathogenic mechanism across diverse tissues and evaluated its potential relevance to peripheral neuropathy.

**Methods:**

Studies were identified in which LPS was the independent exposure and pro-inflammatory, M1-like macrophage activation/polarization was an outcome. Structured evidence mapping was used to code in-vivo studies for direct measurement of prespecified steps along a proposed pathway: gut perturbation→barrier disruption→circulating LPS→systemic inflammation→tissue interface disruption→innate immune activation→M1-like macrophage skew→tissue dysfunction. Conditional concordance and downstream chain completeness scores were calculated.

**Results:**

Mechanistic patterns were conserved between pulmonary, cardiac, renal, lymphatic, gastrointestinal, central nervous, adipose, osseous, urologic, dental, hepatic, uterine, and pancreatic tissues. Conditional concordance with the proposed pathway was high (mean 0.984 ± 0.053). Eleven studies assessed all downstream steps from LPS exposure to tissue dysfunction, each demonstrating full chain completeness. M1 macrophage skew (87%), innate immune activation (87%), and circulating LPS (82.6%) were the most frequently reported steps.

**Conclusions:**

These findings demonstrate conservation of LPS-driven M1-like macrophage polarization and tissue injury across systems, supporting the need to further investigate the biological plausibility of a gut-immune-nerve axis contributing to peripheral neuropathy.

## Background

Gut-organ axes are complex, bidirectional communication networks linking the gastrointestinal tract to major organ systems, including the central nervous system (CNS), endocrine, immune, and pulmonary systems. While gut-organ interdependence has been recognized since the eighteenth century [1], only recently has research begun to elucidate the profound impact that the gut microbiome itself has on systemic human health [2].

The gut microbiome is an intricate ecosystem composed of bacteria, viruses, fungi, archaea, and unicellular eukaryotes. Under homeostatic conditions, these trillions of microorganisms work symbiotically with the human body to modulate host physiology. Beyond its digestive roles, the microbiome also serves as a critical immunologic barrier and biosynthetic hub, producing essential vitamins, short-chain fatty acids, polyamines, polyphenols, and neurotransmitters [3, 4].

Host-microbiome cross-regulation is critical for intestinal homeostasis, supporting beneficial colonization while suppressing pathogenic overgrowth. When this homeostatic environment is disrupted, conditions in the gut are often favorable for gram-negative pathogenic species to flourish [5, 6]. These perturbations in the gut microbial composition (known as “gut dysbiosis”) have been increasingly implicated in diverse pathophysiological states across the human body [7–11]. A critical driver of gut dysbiosis-related pathology is the translocation of gram-negative bacterial endotoxin, lipopolysaccharide (LPS), into the bloodstream [12]. Chronic, sub-acute translocation of LPS is known as metabolic endotoxemia.

Lipopolysaccharide is well characterized as a potent inducer of chronic inflammation, promoting M1-like macrophage polarization and pro-inflammatory cascades throughout the body. As such, LPS-associated cellular damage and dysregulation have already been described in most major organ systems, including the immune privileged environment of the CNS [13–18]. However, LPS-driven macrophage polarization in peripheral nerve tissue remains unexplored.

While more permissive than the CNS, the peripheral nerve microenvironment still exhibits partial immune privilege. As such, the tight junctions comprising the blood-nerve barrier (BNB) are also susceptible to degradation by systemic pro-inflammatory cytokines, such as IL-1*β*, IL-6, and TNF-*α* [19]. Compromise to the BNB may permit infiltration of LPS and other circulating immune mediators, initiating neuroimmune dysregulation, including oxidative stress, demyelination, Schwann cell dysfunction, and eventual neuropathy.

The prevalence of gut dysbiosis continues to rise, driven by increased rates of type 2 diabetes, autoimmune conditions, poor diet, sedentary lifestyle, chronic stress, and cancer therapies [20]. These epidemiologic trends inflate the risk of LPS-mediated systemic inflammation and associated pathologies across tissue types. However, no studies have yet examined whether LPS exposure, secondary to gut dysbiosis and metabolic endotoxemia, directly induces pro-inflammatory macrophage polarization within peripheral nerve tissue.

Therefore, this systematic review synthesizes existing mechanistic data on LPS-induced, pro-inflammatory M1 macrophage polarization across diverse tissues to propose a novel pathophysiologic axis linking gut dysbiosis and peripheral neuropathy.

## Methodology

In order to narrow the search to LPS-driven, M1-mediated tissue dysfunction, the following inclusion criteria were employed: LPS as an independent exposure factor, pro-inflammatory macrophage polarization (or validated proxy markers such as TLR4, NF-κB, MyD88, CD86, iNOS, etc.) as outcome, in-vitro or in-vivo study design. Correspondingly, the exclusion criteria were as follows: abstract-only publications, review articles, absence of LPS exposure, lack of macrophage polarization measurements, non-English language, clinical models, pre-prints. Built-in search filters were applied to include only: “Full-text available”, “English”, “Other animals”, “Exclude pre-prints”.

Following PRISMA recommendations, a structured search was conducted via the PubMed Advanced Search Builder, on April 7, 2025, utilizing the following string:

("lipopolysaccharide" OR "LPS") AND ("macrophage polarization" OR "M1 macrophage") AND ("TLR4" OR "NF-kappaB" OR "MyD88" OR "CD86" OR "iNOS").

Search results were exported as a .csv file and loaded into Rayyan.ai (Rayyan Systems Inc., Cambridge, MA). Rayyan is a web-based, AI-assisted literature review tool which structurally aids in the organization, categorization, and tracking of manuscripts at the time of screening.

To enhance literature coverage, backward citation chaining of included articles was also performed to identify any relevant studies not captured in the primary search.

During screening, a number of studies were excluded despite meeting the basic exposure and outcome criteria. In these cases, LPS was used as an experimental tool to polarize macrophages in studying unrelated processes, rather than as the central focus of mechanistic investigation. These studies were reviewed to inform the methodological landscape but were not included in the final synthesis.

The model for binary, M1/M2 polarization of macrophages has undergone conceptual updates in recent years to reflect nuances in intermediate macrophage activation between the two terminal states [21]. This paradigm shift in biological understanding became widely accepted in the mid- to late-2010s. However, as this review was designed to evaluate mechanistic pathways from a wide variety of studies, with no sanctions placed on the year of their respective publication, “M1” or “M1-like” macrophage definitions were used to ensure consistency and tractability of analysis between studies.

Given heterogeneity across tissues, models, and endpoints, effect-size pooling was not appropriate. We therefore performed a structured evidence-mapping synthesis by coding each study for direct measurement of prespecified mechanistic steps along the proposed pathway. Steps were coded as binary indicators (0, 1) and summed to generate a chain-completeness score reflecting how many mechanistic elements were empirically supported per study. Studies were additionally categorized by exposure class (endogenous vs. exogenous endotoxin exposure) to distinguish etiologic plausibility from mechanistic sufficiency. Because several pathway elements (circulating LPS, systemic inflammation, barrier/interface disruption, and organ-level dysfunction) are not assessable in-vitro, chain-completeness scoring was restricted to in-vivo studies.

## Results

### Study overview

**Fig. 1:**
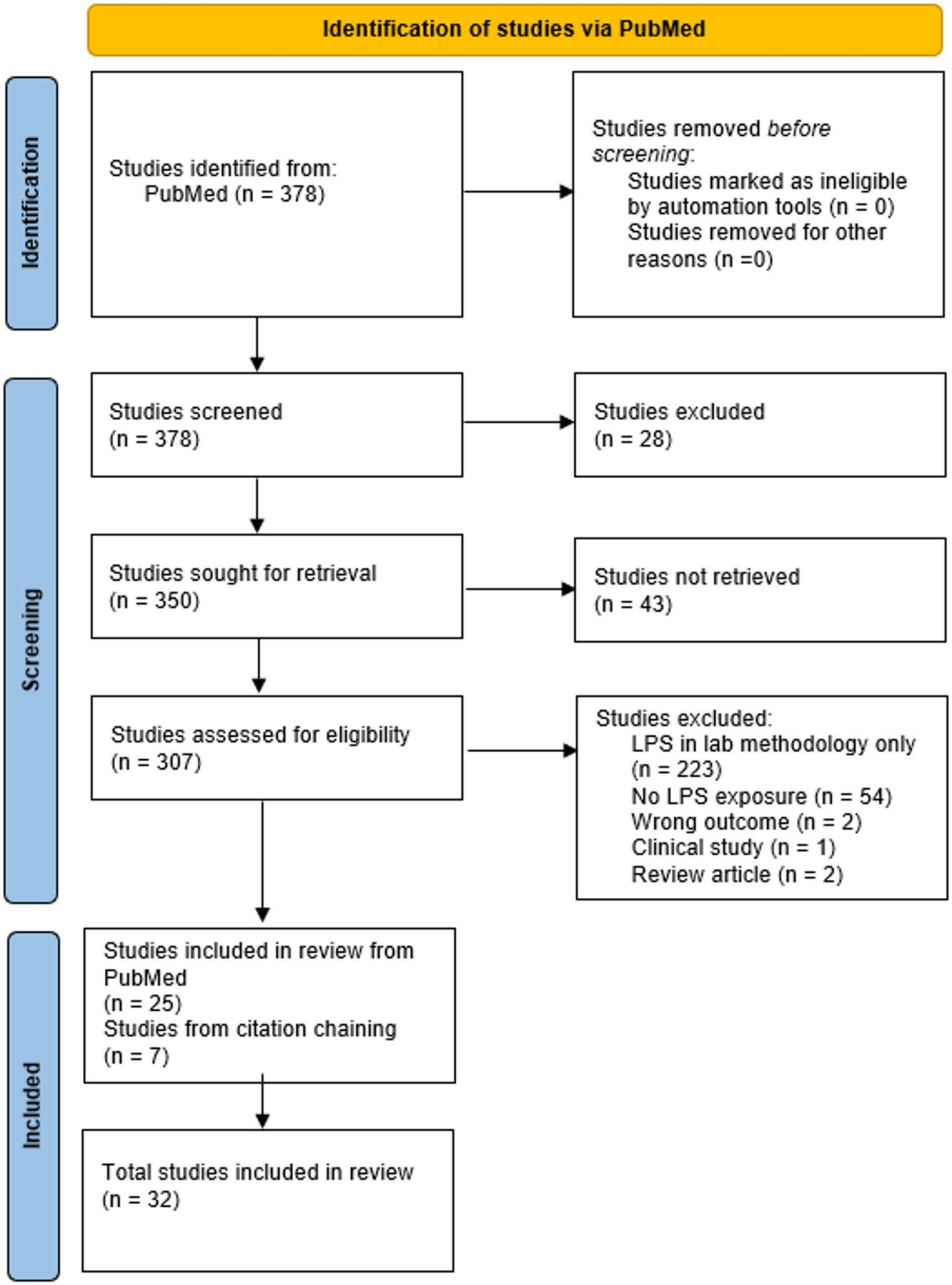
PRISMA flow diagram for study selection process

The PubMed search results yielded 378 studies; ultimately 32 met criteria to be included in narrative review. Studies were published from 2003 to 2024, utilized murine models (50%), in-vitro models (22%), or a combination (28%); with investigational scope which covered molecular mechanisms, immunologic pathways, and therapeutic strategies. Reported tissue types included pulmonary, cardiac, renal, lymphatic, gastrointestinal, nervous (brain), adipose, osseous, urologic, dental, hepatic, pancreatic, and uterine. Common inflammatory cells and markers included, M1 macrophage counts, nitric oxide (NO)/inducible nitric oxide synthase (iNOS), TNF-*α*, IL-6, IL-1*β*, MyD88, and NF-κB. In animal models, metabolic endotoxemia was induced via direct LPS injection, antibiotic courses or fatty diets to trigger gut dysbiosis, or cecal ligation and puncture. The methodological landscape included transcriptomics, cytokine profiling, and disease progression models (Fig. 1, Table 1).

**Table 1:** Summary of studies included in review, organized by organ/system investigated by each author group

Organ/system	Lead author	Year	Title	Type of investigation	Model(s)	Mechanistic findings	Proposed pathway conservation?
Cardiac	Dai	2015	Epoxyeicosatrienoic acids regulate macrophage polarization and prevent LPS-induced cardiac dysfunction	Therapeutic	In-vitro	This study investigates the role of epoxyeicosatrienoic acids in regulating macrophage polarization and mitigating LPS-induced cardiac dysfunction. EETs significantly inhibited M1 macrophage polarization and pro-inflammatory cytokine production while promoting M2 polarization and IL-10 expression. Mechanistically, EETs downregulated NF-κB activation, reducing cardiac dysfunction and M1 macrophage infiltration post-LPS treatment	Yes—LPS-induced M1 macrophage polarization and cardiac dysfunction tied to TLR4/NF-κB pathways
	Liang	2020	Interleukin-5 deletion promotes sepsis-induced M1 macrophage differentiation, deteriorates cardiac dysfunction, and exacerbates cardiac injury via the NF-κB p65 pathway in mice	Therapeutic	Murine	This study investigates the role of IL-5 in sepsis-induced cardiac injury using a LPS mouse model. Findings reveal that IL-5 deficiency exacerbates cardiac dysfunction, increases myocardial apoptosis, and promotes M1 macrophage differentiation through the NF-κB p65 pathway	Yes—LPS-mediated M1 macrophage polarization and TLR4/NF-κB signaling is associated with cardiac cell damage
	Xiao	2021	Ferrostatin-1 alleviates lipopolysaccharide-induced cardiac dysfunction	Therapeutic	Murine	This study investigates the effects of Fer-1 on LPS-induced cardiac dysfunction in rats, revealing that Fer-1 significantly improves cardiac systolic function, as evidenced by increased left ventricular ejection fraction and fractional shortening, while reducing serum markers of cardiac injury	Yes—LPS-mediated M1 macrophage polarization and subsequent induction of TLR4/NF-κB signaling pathways contribute to cardiac dysfunction during sepsis
	Wang	2021	Mono-macrophage-derived MANF alleviates bacterial myocarditis by inhibiting NF-kappaB activation and myocardial inflammation	Therapeutic	Murine	This study investigates the role of mono-macrophage-derived mesencephalic astrocyte-derived neurotrophic factor (MANF) in bacterial myocarditis, utilizing an LPS-induced mouse model. Findings reveal that MANF deficiency in macrophages exacerbates myocardial inflammation	Yes—LPS-mediated M1 macrophage polarization is noted in myocardial tissue via NF-κB pathways
	Zhu	2019	Thyroxine affects lipopolysaccharide-induced macrophage differentiation and myocardial cell apoptosis via the NF-κB p65 pathway both in vitro and in vivo	Therapeutic	Murine, In-vitro	This study investigates the role of thyroxine in mitigating LPS-induced myocardial cell apoptosis through modulation of macrophage differentiation and the NF-κB p65 pathway	Yes—LPS-mediated M1 macrophage polarization was shown to exacerbate cardiac dysfunction and cell apoptosis
CNS	Qin	2007	Systemic LPS causes chronic neuroinflammation and progressive neurodegeneration	Mechanistic	Murine	Study of long-term effects of a single systemic LPS injection on neuroinflammation in adult mice. Following LPS administration, brain TNF-*α* levels remained elevated for up to 10 months, while peripheral TNF-*α* levels returned to baseline within a week. The study demonstrates that LPS-induced TNF-*α* activates microglia, leading to chronic neuroinflammation and a progressive loss of dopaminergic neurons in the substantia nigra. Suggests inflammation can trigger a self-perpetuating cycle of neuroinflammation and neurodegeneration	Yes—systemic LPS injection contributed to neuroinflammation in the CNS
	Wang	2019	Geraniin attenuates lipopolysaccharide-induced cognitive impairment in mice by inhibitingtyoll-like receptor 4 activation	Therapeutic	Murine	This study investigates the neuroprotective effects of geraniin, a polyphenolic compound, against LPS-induced cognitive impairment in mice. The therapeutic strategy mitigated LPS-induced pro-inflammatory cytokine release	Yes—LPS-mediated M1 macrophage polarization was shown to contribute to neuroinflammation
	Fan	2020	Interferon regulatory factor 5 mediates lipopolysaccharide-induced neuroinflammation	Therapeutic	Murine, In-vitro	This study investigates the role of Interferon Regulatory Factor 5 (IRF5) in LPS-induced neuroinflammation. Using C57/BL6 mice and BV2 microglial cells, the study found that LPS significantly elevated IRF5 expression in mouse brains, co-localizing with activated microglia	Yes—LPS activated microglia in the brain via mechanisms similar to peripheral M1 macrophage polarization
Dental	Choe	2020	Effect of nifedipine, a calcium channel blocker, on the generation of nitric oxide and interleukin-1*β* by murine macrophages activated by lipopolysaccharide from Prevotella intermedia	Therapeutic	In-vitro	This study investigates the effects of nifedipine, a calcium channel blocker, on nitric oxide (NO) and IL-1*β* production in murine macrophages activated by LPS from *Prevotella intermedia*, a periodontal pathogen	Yes—LPS-mediated M1 macrophage polarization was implicated in the production of pro-inflammatory products in periodontal pathology
GI	Zhu	2016	Baicalin ameliorates experimental inflammatory bowel disease through polarization of macrophages to an M2 phenotype	Therapeutic	Murine, In-vitro	In this therapeutic investigation, in vitro baicalin inhibited LPS-induced M1 polarization, decreasing pro-inflammatory cytokines (TNF-*α*, IL-23)	Yes—LPS-induced macrophage polarization was associated with pro-transcription factors and cytokine profiles
	Kang	2023	L-arabinose attenuates LPS-induced intestinal inflammation and injury through reduced M1 macrophage polarization	Therapeutic	Murine, in-vitro	This study investigates the anti-inflammatory effects of L-arabinose in LPS-induced intestinal inflammation model using male C57BL/6 mice. L-arabinose supplementation significantly improved intestinal morphology, reduced serum levels of pro-inflammatory cytokines (TNF-*α*, IL-1*β*, IL-6), and increased tight junction proteins (occludin, claudin-1) compared to the LPS group	Yes—LPS mediated M1 macrophage polarization was associated with NF-κB pathway activation and pro-inflammatory cytokine release in the intestine
Immune	Das	2018	High-resolution mapping and dynamics of the transcriptome, transcription factors, and transcription co-factor networks in classically and alternatively activated macrophages	Mechanistic	In-vitro	A transcriptomic study elucidating the transcription dynamics in LPS-induced M1 macrophages versus IL-4/IL-13-induced M2 macrophages. LPS-stimulated bone marrow-derived macrophages revealed significantly enriched binding sites for NF-κB group transcription factors, downstream of TLR4-LPS binding	Yes—LPS-mediated M1 macrophage polarization demonstrated unique transcription dynamics downstream of TLR4/NF-κB signaling
Lymphatic	Chakraborty	2015	Lipopolysaccharide modulates neutrophil recruitment and macrophage polarization on lymphatic vessels and impairs lymphatic function in rat mesentery	Mechanistic	Murine	This study investigates the impact of LPS on lymphatic function and innate immune cell dynamics in rat mesentery. LPS activated inflammatory pathways in lymphatic muscle cells via TLR4. These findings suggest that LPS-induced inflammation impairs lymphatic function through immune cell modulation and cytokine dysregulation	Yes—LPS-mediated M1 macrophage polarization dysregulated lymphatic tissues via TLR4 activation and pro-inflammatory cytokine production
Metabolic	Liddle	2020	CD8( +) T cell/adipocyte inflammatory cross talk and ensuing M1 macrophage polarization are reduced by fish-oil-derived n-3 polyunsaturated fatty acids, in part by a TNF-*α*-dependent mechanism	Therapeutic	In-vitro	The study demonstrates that fish oil-derived long-chain n-3 polyunsaturated fatty acids significantly reduce inflammatory cross-talk between CD8 + T cells and adipocytes, leading to decreased M1 macrophage polarization	Yes—LPS-mediated M1 macrophage polarization can occur via immune cell cross-talk, resulting in pro-inflammatory cytokine release
	Zhao	2018	Suppression of TLR4 by miR-448 is involved in diabetic development via regulating macrophage polarization	Mechanistic	Murine	In diabetic mouse models, LPS was shown to induce M1 macrophage polarization, characterized by increased levels TNF-*α*, IL-6, and iNOS, alongside increased TLR4 expression. Systemic inflammation induced by LPS-mediated macrophage polarization can exacerbate beta cell death and dysfunction in diabetics	Yes—Systemic LPS-mediated M1 macrophage polarization was associated with increased TLR4, pro-inflammatory pathways
	Li	2024	Gut dysbiosis contributes to SCFAs reduction-associated adipose tissue macrophage polarization in gestational diabetes mellitus	Mechanistic	Murine, in-vitro	TLR4 mRNA expression was elevated in the adipose tissue of mice on a high-fat diet and those receiving fecal microbiota transplants from gestational diabetes mellitus patients. In vitro experiments further confirmed this, showing that LPS treatment directly induced the TLR4-NF-κB signaling pathway in macrophage cells	Yes—LPS-mediated M1 macrophage polarization was upregulated in both murine model types, as well as in in-vitro work
Microbiologic	Wang	2021	Lipopolysaccharide from biofilm-forming Pseudomonas aeruginosa PAO1 induces macrophage hyperinflammatory responses	Mechanistic	In-vitro	This study investigates the effects of LPS from biofilm-forming *Pseudomonas aeruginosa* PAO1 on macrophage polarization. The results demonstrate that LPS from biofilm-forming PAO1 induces significantly stronger hyperinflammatory responses in human THP-1 and murine Raw264.7 macrophages compared to LPS from planktonic PAO1, correlating with increased MyD88 expression	Yes—LPS-mediated M1 macrophage polarization and inflammatory cascades may be stronger in bacterial colonies which form biofilm vs planktonic bacterium
Mixed	Xu	2016	Isomeranzin suppresses inflammation by inhibiting M1 macrophage polarization through the NF-κB and ERK pathway	Therapeutic	Murine, In-vitro	This study demonstrated that isomeranzin inhibited M1 macrophage polarization through the NF-κB and ERK signaling pathways. The study demonstrated that isomeranzin reduced pro-inflammatory cytokines IL-1*β* and IL-6 in LPS-activated macrophages	Yes—LPS-induced M1 macrophage polarization and associated TLR4/NF-κB pro-inflammatory pathways were attenuated via treatment
Obstetric	Elovitz	2003	A New model for inflammation-induced preterm birth	Mechanistic	Murine	This study presents a novel mouse model for localized intrauterine inflammation, associated with 100% pre-term delivery without maternal mortality. The model used LPS infusion into gestation sacs to trigger M1 macrophage polarization and localized inflammation	Yes—LPS-mediated M1 macrophage polarization was used to trigger intrauterine inflammation via TLR4/NF-κB pathways
	Agrawal	2015	Role of notch signaling during lipopolysaccharide-induced preterm labor	Mechanistic	Murine	This study investigates the role of Notch signaling in LPS-induced preterm labor in mice. LPS-treated decidual macrophages secreted higher levels of M1-associated pro-inflammatory cytokines, such as IL-1*β*, TNF-*α*, and IL-6	Yes—LPS-induced intrauterine injection led to decidual macrophages producing M1/TLR4 associated pro-inflammatory cytokines
Orthopedic	Wang	2023	Osthole inhibits M1 macrophage polarization and attenuates osteolysis in a mouse skull model	Therapeutic	Murine	The study investigates the effects of osthole (OST) on M1 macrophage polarization and osteolysis in a LPS-induced mouse skull model. OST significantly suppressed LPS-induced M1 markers (iNOS) while enhancing M2 markers	Yes—LPS-mediated M1 macrophage polarization was associated with pro-inflammatory cytokine release via TLR4/NF-κB pathways in osseous tissue
Pulmonary	Sul	2021	Quercetin prevents LPS-induced oxidative stress and inflammation by modulating NOX2/ROS/NF-kB in lung epithelial cells	Therapeutic	In-vitro	The study investigates the protective effects of quercetin against LPS-induced oxidative stress and inflammation in human lung epithelial A549 cells. Quercetin suppressed the nuclear translocation of NF-κB and decreased pro-inflammatory cytokines TNF-*α*, IL-1*β*, and IL-6 following LPS stimulation	Yes—LPS-mediated M1 macrophage polarization demonstrated an increase of pro-inflammatory cytokine production in lung tissue via NF-κB pathways
	Wang	2017	A20 protein regulates lipopolysaccharide-induced acute lung injury by downregulation of NF-κB and macrophage polarization in rats	Therapeutic	Murine	The study investigates the role of A20 protein in regulating LPS-induced acute lung injury in rats, focusing on its effects on the NF-κB signaling pathway and macrophage polarization. Elevated levels of TNF-*α* and IL-1*β* were observed in LPS-treated macrophages	Yes—LPS induced M1 macrophage polarization and TLR4/NF-κB signaling was observed in acute lung injury and attenuated via inhibition of NF-κB DNA
	Zhang	2021	Loganin alleviates sepsis-induced acute lung injury by regulating macrophage polarization and inhibiting NLRP3 inflammasome activation	Therapeutic	Murine	The study investigates the anti-inflammatory effects of loganin, an iridoid glycoside from *Corni fructus*, in a murine model of sepsis-induced acute lung injury. Mechanistically, loganin inhibited M1 macrophage polarization while promoting M2 polarization, and suppressed NLRP3 inflammasome activation via ERK and NF-κB signaling pathways	Yes—LPS-mediated M1 macrophage polarization was shown in a sepsis model to increase pro-inflammatory pathways via ERK and NF-κB signaling pathways
	Zhang	2020	LincRNA-p21 promotes classical macrophage activation in acute respiratory distress syndrome by activating NF-κB	Mechanistic	Murine, In-vitro	Acute Respiratory Distress Syndrome was induced in mice via intratracheal administration of LPS; alveolar macrophages were treated with LPS. LPS was noted to induce M1 activation via NF-κB pathways and p65 nuclear translocation. The pro-inflammatory process was also mediated by lincRNA-p21	Yes—LPS-mediated M1 macrophage polarization noted in lung tissue and in-vitro alveolar macrophages
	Zhou	2022	Polygonatum polysaccharide regulates macrophage polarization and improves LPS-induced acute lung injury through TLR4-MAPK/NF-κB pathway	Therapeutic	Murine, In-vitro	In an LPS-induced ALI mouse model, PSPs reduced total protein levels and inflammatory cell counts in bronchoalveolar lavage fluid, alongside downregulating inflammatory markers. The mechanism involves the suppression of the TLR4-MAPK/NF-κB signaling pathway	Yes—LPS-mediated M1 macrophage polarization is shown to upregulate TLR4-MAPK/NF-κB signaling pathways in acute lung injury
	Qiu	2019	Modulation of intestinal microbiota by glycyrrhizic acid prevents high-fat diet-enhanced pre-metastatic niche formation and metastasis	Therapeutic	Murine	This study investigates therapeutic strategies to attenuate LPS-induced inflammation pathways which may contribute to the formation of metastatic niches	Yes—LPS-induced M1 macrophage polarization and subsequent TLR4/NF-κB inflammation pathways are shown to contribute to the formation of metastatic niches
Renal	Cui	2023	DsbA-L deletion attenuates LPS-induced acute kidney injury by modulating macrophage polarization	Therapeutic	Murine	In the study DsbA-L deletion was shown to attenuate LPS-induced acute kidney injury in a mouse model, evidenced by significantly lower serum creatinine levels and reduced inflammatory cytokines, particularly IL-6. DsbA-L knockout led to decreased M1 macrophage polarization and downregulated NF-κB/AP-1 signaling	Yes—LPS-mediated M1 macrophage polarization was associated with pro-inflammatory TLR4/NF-κB pathways in renal tissue
Sepsis	Sawoo	2021	TLR4 and TNFR1 blockade dampen M1 macrophage activation and shifts them towards an M2 phenotype	Therapeutic	In-vitro	The study investigates the effects of dual blockade of TLR4 and TNFR1 on LPS-induced M1 macrophage activation. The blockade resulted in decreased levels of pro-inflammatory cytokines TNF-*α* and IL-1*β*, reduced oxidative stress markers, and increased antioxidant enzyme activities. Findings show strategy for managing sepsis by promoting M1 to M2 macrophage switching, thereby mitigating inflammation and oxidative damage	Yes—LPS-mediated M1 macrophage polarization is well known as driver of septic shock via initiation of NF-κB signaling pathways and pro-inflammatory cytokine release
Sepsis	Li	2022	Trichinella spiralis cystatin alleviates polymicrobial sepsis through activating regulatory macrophages	Therapeutic	Murine	This study investigates the therapeutic effects of recombinant Trichinella spiralis cystatin (rTs-Cys) on polymicrobial sepsis in BALB/c mice. Administering rTs-Cys significantly improved survival rate. Mechanistically, rTs-Cys shifted macrophage polarization from M1 to M2 by inhibiting the TLR2/MyD88	Yes—LPS-mediated M1 macrophage polarization was associated with pro-inflammatory cytokine release via TLR4/NF-κB pathways in sepsis
Systemic	Lee	2024	LPS-induced systemic inflammation is suppressed by the PDZ motif peptide of ZO-1 via regulation of macrophage M1/M2 polarization	Therapeutic	Murine	This study investigates the therapeutic potential of PEGylated PDZ peptides derived from ZO-1 in mitigating LPS-induced systemic inflammation. Therapy downregulated TLR4/NF-κB signaling and associated pro-inflammatory cytokine release, reducing tissue damage across multiple organ systems	Yes—LPS-mediated M1 macrophage polarization in the context of systemic inflammation was modeled, demonstrating TLR4/NF-κB signaling and associated pro-inflammatory cytokine release
Urologic	Fang	2022	TcpC inhibits M1 but promotes M2 macrophage polarization via regulation of the MAPK/NF-κB and Akt/STAT6 pathways in urinary tract infection	Therapeutic	Murine, In-vitro	This study investigates TcpC, a virulence factor of uropathogenic *Escherichia coli*, and its role in macrophage polarization during urinary tract infections. TcpC significantly inhibits M1 markers (CD80, CD86, iNOS) while promoting M2 markers (CD163, CD206, Arg-1) in both in vivo and in vitro models. Mechanistically, TcpC down-regulates the MAPK/NF-κB pathway (p38, ERK, p50, p65) and up-regulates the Akt/STAT6 pathway, thereby favoring M2 polarization	Yes—LPS-mediated M1 macrophage polarization is associated with MAPK/NF-κB pathway activation and pro-inflammatory cytokine release in the urinary tract

### M1 Macrophage polarization mechanism

The core mechanism underlying LPS-mediated macrophage polarization and tissue dysfunction is conserved across diverse organ systems, including pulmonary [22–27], cardiac [28–32], renal [33], lymphatic [34], gastrointestinal [35, 36], nervous (brain) [14, 37, 38], adipose [39, 40], osseous [41], urologic [42], dental [43], hepatic [44], uterine [45, 46], pancreatic [47], and global/systemic [48–52] (Table 1).

In the studies evaluated, LPS originated from endogenous gut sources or exogenous injection; regardless of endotoxin origin, similar innate immune pathways were triggered. Across tissue types, the M1 macrophage polarization mechanism begins with the recognition of the LPS bioactive lipid A domain. This moiety binds either soluble or membrane-bound lipopolysaccharide-binding protein (LBP), forming an LPS-LBP complex that is transferred to CD14. The CD14 protein acts as a co-receptor, presenting the LPS ligand to the transmembrane pattern recognition receptor, Toll-like receptor 4 (TLR4), which is universally expressed on macrophage membranes throughout the body.

Toll-like receptor 4’s affinity for LPS is enhanced by its non-covalently associated co-receptor MD-2, which forms a hydrophobic binding pocket for lipid A. After ligand engagement, the TLR4/MD-2 complex dimerizes, triggering downstream signaling via both MyD88-dependent (early-phase NF-κB and MAPK activation) and TRIF-dependent (late-phase IRF3/7 activation and Type I interferon production) pathways. Lipopolysaccharide is demonstrated to be one of the only ligands which concurrently triggers both MyD88 and TRIF pathways.

**Fig. 2:**
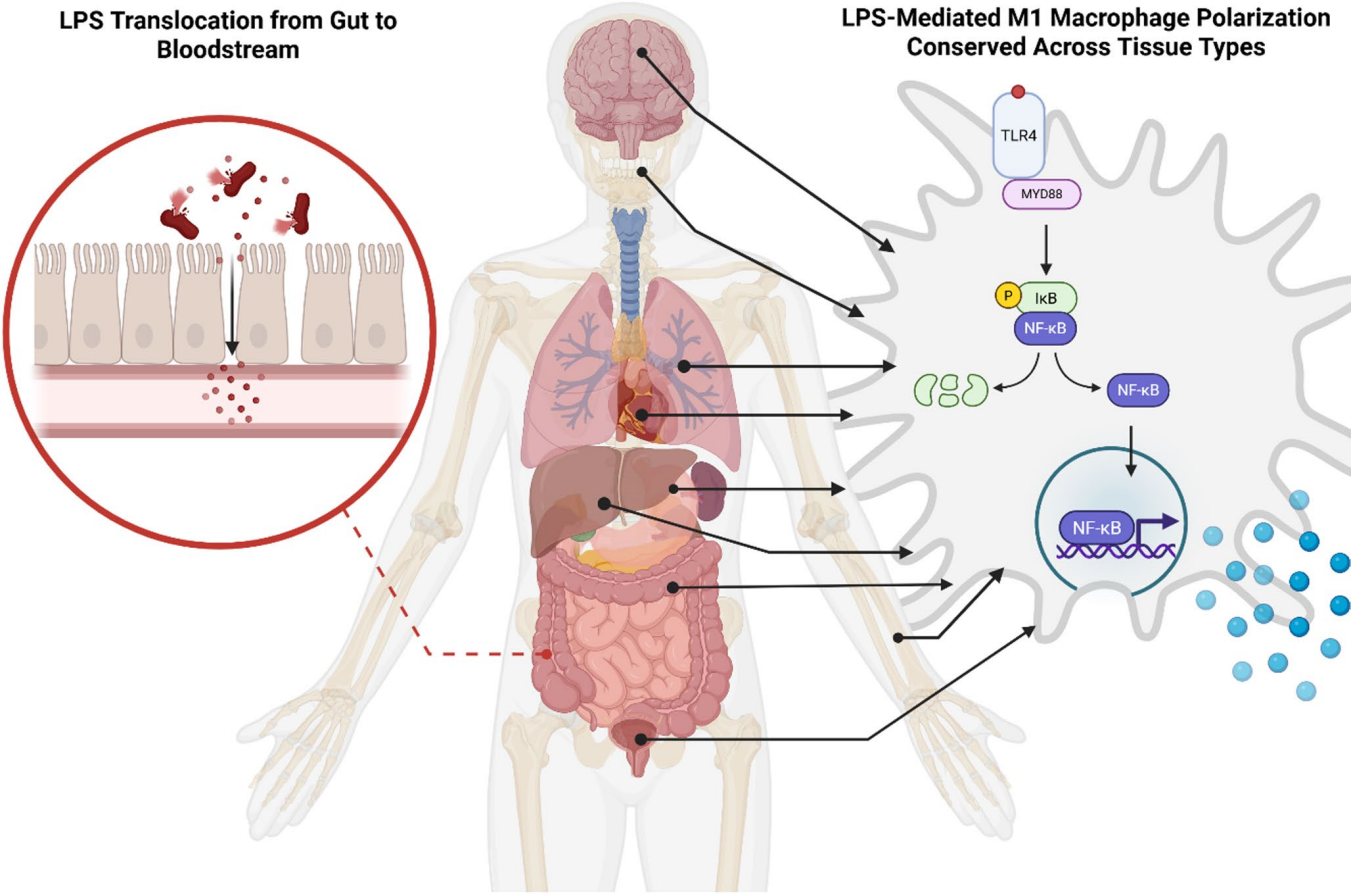
Gut dysbiosis leads to the weakening of tight junctions between epithelial cells in the large intestine. A compromised gut barrier allows for the translocation of LPS into the bloodstream. Across tissue types, circulating LPS binds to TLR4 on the surface of macrophages, triggering NF-κB pathways. This molecular cascade promotes macrophage polarization to a pro-inflammatory (M1-like) phenotype, and upregulates TNF-*α*, IL-6, IL-12, IL-1*β* cytokine production. Chronic innate immune activation is associated with dysfunction across diverse tissue types. This mechanism is conserved throughout the body (original figure created using BioRender software (Toronto, ON, Canada))

Throughout the body, activation of the NF-κB pathway results in macrophage polarization to the classically activated, pro-inflammatory (M1) phenotype, which includes the upregulation of pro-inflammatory cytokines TNF-*α*, IL-6, IL-1*β*, as well as iNOS, and reactive oxygen species (ROS). This inflammation is shown to cause pathophysiologic systemic changes, as well acute disruption of tissues throughout the body. The inflammatory-immune-tissue injury cascade, beginning with the proximate step of LPS from the gut, is shown in Fig. 2.

### Mechanistic chain completeness evaluation

The author-proposed mechanism linking gut dysbiosis to peripheral neuropathy mirrors pathways previously demonstrated across multiple organ systems. To quantify this mechanistic conservation across heterogeneous models, we constructed an evidence map for in-vivo studies capturing direct measurement of the following key pathway steps:

(1) Gut perturbation→(2) gut barrier disruption→(3) circulating LPS→(4) systemic inflammatory response→(5) tissue interface disruption→(6) tissue innate immune activation→(7) M1-like macrophage skew→(8) tissue dysfunction.

If a pathway step was explicitly evaluated in a study and exhibited positive findings, it was scored as “1”; if a step was explicitly evaluated in a study and exhibited null or negative findings it was scored as “0”. To mitigate the risk of false positive or negative bias in mechanistic concordance or completeness, no evidentiary contribution was assigned to mechanistic steps not evaluated in the included literature. Therefore, if a step from the proposed pathway was not examined in an included study, it was marked as “not evaluated” and removed from concordance or completeness calculation. For studies using gut dysbiotic models as a source of endogenous LPS, scoring began at gut perturbation; for studies using injected exogenous LPS, scoring began at the most proximal assessable step (either circulating LPS or tissue innate immune activation, depending on study design). A study was considered to exhibit full downstream chain completeness only if all steps from proximal LPS introduction through tissue dysfunction were individually evaluated and positive.

Twenty-three in-vivo studies were eligible for mechanistic mapping. Eleven studies explicitly evaluated all downstream mechanistic steps from LPS introduction to tissue dysfunction; all 11 (100%) exhibited full downstream chain completeness. Across all in-vivo studies, we additionally calculated conditional concordance (number of positive steps divided by number of steps assessed). Mean conditional concordance was high among the included studies (0.984 ± 0.053; range 0.80–1.00), indicating strong alignment of assessed findings with the proposed pathway.

Among downstream steps, the most frequently evaluated were M1 macrophage skew (20/23, 87.0%), tissue innate immune activation (20/23, 87.0%), and circulating LPS (19/23, 82.6%). Direct evidence of tissue interface disruption associated with LPS was assessed in 15 studies, all of which reported positive findings (100%).

Together, this evidence map indicates that, following systemic LPS introduction, the downstream inflammatory-immune-tissue injury cascade demonstrates conservation across diverse models, organ systems evaluated, and irrespective of whether LPS exposure was endogenous or exogenous.

### Tissue-specific nuances

While the core molecular cascade by which LPS polarizes macrophages toward an M1-like phenotype demonstrates conservation regardless of tissue type, the expression of this polarization may be further modulated by tissue-specific microenvironments, macrophage ontogeny, and local immunologic signaling. As such, some tissues exhibit a modified M1 response correspondent with either increased sensitivity or tolerogenic demands.

#### Alveolar macrophages

Alveolar macrophages (AMs), located throughout the alveolar epithelial layer at the air-tissue interface, are chronically exposed to airborne pathogens, allergens, and particulates. To prevent excessive inflammation and preserve the integrity of the alveolar gas-exchange barrier, AMs exhibit a tightly regulated pro-inflammatory polarization response.

The immune restraint of AMs is maintained, in part, through cross-talk with alveolar epithelial cells, particularly type II pneumocytes, which secrete immunomodulatory signals including granulocyte–macrophage colony-stimulating factor, transforming growth factor-beta, and surfactant proteins A and D. These factors work in concert to preserve AM homeostasis and suppress inappropriate TLR activation. However, with LPS exposure, regulatory control mechanisms become overwhelmed, driving AMs largely toward a pro-inflammatory, M1-like phenotype.

The resultant pro-inflammatory environment can disrupt epithelial integrity and promote alveolar-capillary barrier breakdown. This pathophysiology is a hallmark of acute lung injury and acute respiratory distress syndrome, both of which are frequently observed in septic patient populations.

#### Adipose macrophages

Adipose tissue, particularly in the context of clinical obesity, is actively inflammatory. As such, infiltrating macrophages within adipose tissue are exposed to a pro-inflammatory microenvironment conducive to M1 phenotyping.

Adipocytes and macrophages can synergistically trigger pro-inflammatory cascades in one another, which often results in a feed-forward inflammatory loop, especially in the presence of LPS. For instance, LPS can intensify adipocyte release of monocyte chemoattractant protein-1 and serum amyloid A. Both of these factors act as chemoattractants, recruiting more monocytes and promoting M1 polarization, which increases localized levels of pro-inflammatory cytokines. Additionally, LPS itself triggers lipolysis in adipocytes, increasing levels of free fatty acids (FFA). Like LPS, the FFA molecule is a ligand which activates TLR4 pathways. Lipopolysaccharide-liberated FFAs therefore increase M1 macrophage polarization alongside LPS, further amplifying pro-inflammatory cytokine production, accelerating lipolysis, and sustaining a self-perpetuating inflammatory loop. It is this inflammatory microenvironment in adipose tissue that also contributes significantly to systemic low-grade inflammation and metabolic dysfunction in obesity [39, 40].

#### Microglia

The CNS maintains an immune-privileged, anti-inflammatory microenvironment, tightly regulated by cross-talk among astrocytes, neurons, and microglia. Homeostasis is maintained by immunomodulatory cytokines such as TGF-*β*, interleukin-10 (IL-10), and CX3CL1 (fractalkine).

Unlike peripheral macrophages, microglia are long-lived, yolk-sac derived cells with tightly restricted activation profiles under baseline conditions. Upon systemic exposure to LPS, the resultant elevated levels of circulating cytokines act in concert with LPS to disrupt the integrity of the blood-brain barrier (BBB) by degrading tight junction proteins (e.g., ZO-1, occludin, claudins) and upregulating leukocyte adhesion molecules (ICAM-1, VCAM-1). This barrier compromise permits the infiltration of peripheral immune cells, plasma proteins, and microbial products into the CNS parenchyma.

Upon BBB disruption, microglia, alongside astrocytes, produce ROS, NO, and pro-inflammatory cytokines (e.g., IL-1*β*, TNF-*α*), amplifying neuroinflammation. This self-sustaining immune activation loop contributes to further BBB dysfunction, synaptic damage, and neuronal injury. Chronic dysregulation of this axis has been implicated in the progression of neurodegenerative and neuropathic disorders. Notably, these mechanisms underscore the vulnerability of glial-regulated, barrier-protected tissues to systemic immune disruptions, parallels that will be further explored in the context of peripheral nerve tissue.

### Proposed LPS-driven peripheral neuropathy

The BNB is dual-layered, composed of the endothelial tissue of intrinsic nerve microvasculature and the multilayered perineurium which encases nerve fascicles. This selectively permeable barrier provides structural protection and maintains an immune-privileged endoneurial microenvironment surrounding peripheral nerve axons and their associated glial support cells.

A stepwise pathophysiologic link between gut dysbiosis and BNB compromise has been increasingly delineated in the literature. Mechanisms have been described for the disruption of gut epithelial tight junctions [53], which allows translocation of bacterial LPS into the bloodstream [54]. Circulating LPS induces systemic inflammation through activation of TLR4 on monocytes and macrophages, driving secretion of pro-inflammatory cytokines such as TNF-*α*, IL-1*β*, and IL-6 [55]. These cytokines, alongside LPS itself, are known to degrade endothelial tight junctions and upregulate leukocyte adhesion molecules [56], including within the vasculature of the BNB [57]. As such, it is biologically plausible that systemic inflammation may result in increased permeability of the peripheral nerve barrier, facilitating infiltration of circulating immune cells, cytokines, and microbial products into the otherwise immune-restricted nerve tissue.

This stepwise progression from epithelial gut barrier dysfunction to neurovascular compromise outlines a feasible mechanistic pathway from gut dysbiosis to BNB disruption. This is effectively, a “leaky gut to leaky nerve” cascade.

**Fig. 3:**
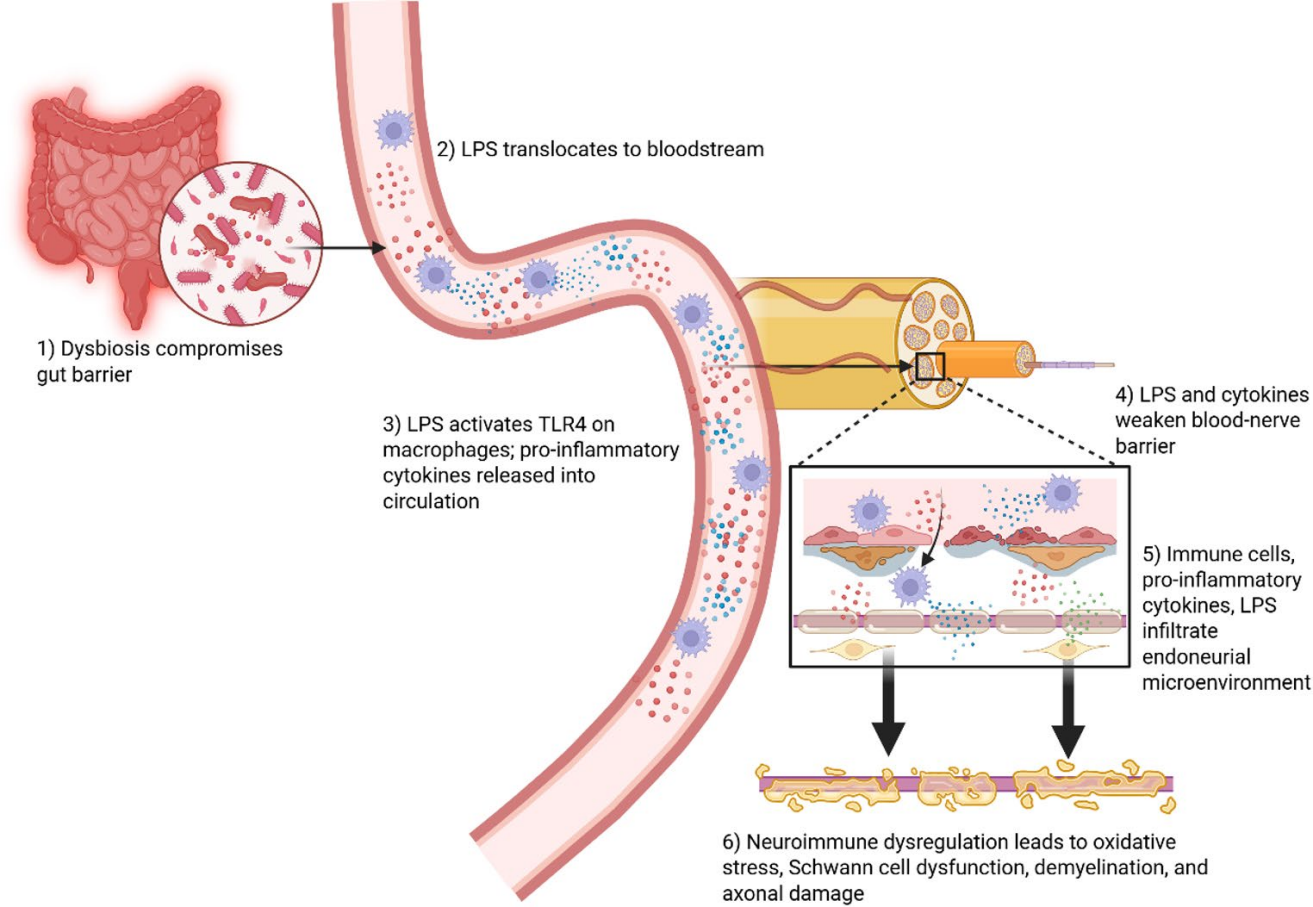
Proposed pathway linking gut dysbiosis to downstream peripheral neuropathy. Original figure created using BioRender software (Toronto, ON, Canada)

Given the conserved nature of LPS-induced M1-like macrophage polarization across diverse tissue types, and the parallels between the CNS and peripheral nerve in terms of barrier regulation, glial support, and immune privilege, the authors propose the following mechanism linking chronic gut dysbiosis to downstream peripheral neuropathy (Fig. 3):

1. Gut dysbiosis – characterized by metabolite imbalance, increased pathogenic bacterial endotoxins (i.e., LPS), thinning of the protective mucus layer, and marked increase in pro-inflammatory cytokines – impairs the integrity of tight junctions between intestinal epithelial cells.
2. Weakened tight junctions and damaged gut epithelial cells allow for increased translocation of LPS into the bloodstream.
3. LPS activates TLR4 on the surface of monocytes and macrophages, triggering NF-κB pathways and upregulating the release of pro-inflammatory cytokines (TNF-*α*, IL-6, IL-1 *β*) in circulation.
4. Increased levels of LPS and pro-inflammatory cytokines access and act upon the intrinsic microvasculature of peripheral nerves and begin to weaken the tight junctions of the endothelial cells of the blood-nerve barrier.
5. The blood-nerve barrier becomes structurally compromised and immune cells, pro-inflammatory cytokines, and LPS infiltrate the immune privileged endoneurial environment surrounding nerve axons.
6. Infiltrating LPS and pro-inflammatory cytokines begin to polarize resident endoneurial macrophages to an M1 or pro-inflammatory phenotype. LPS exposure also changes the functionality of Schwann cells, further inducing pro-inflammatory pathways and amplifying neuroimmune dysregulation. Disruption of homeostatic mechanisms leads to myelin destruction, axonal damage, and neuropathic dysfunction.

It is important to note that Schwann cells play a central role in peripheral nerve immune modulation. Somewhat analogous to glial cells in the CNS, Schwann cells support axonal function and contribute to the maintenance of the BNB. Under homeostatic conditions, Schwann cells help preserve the immune-privileged endoneurial environment through expression of anti-inflammatory mediators and tight junction proteins. However, in the presence of systemic inflammation and LPS infiltration, Schwann cells become reactive, upregulating TLR4, producing pro-inflammatory cytokines themselves (e.g., TNF-*α*, IL-6), and initiating complement cascade activation [58, 59]. These changes, in concert with M1 macrophage infiltration, can drive a pathologic neuroimmune feedback loop that disrupts axonal support, promotes demyelination, and accelerates neuropathic degeneration.

This reactive profile may mirror glial dysregulation in the CNS, highlighting the shared vulnerability of glial-governed, barrier-protected tissues to systemic immunologic perturbation. The parallels between microglial and Schwann cell response pathways further support the plausibility of LPS-driven neuropathy within peripheral nerve tissue under chronic inflammatory conditions.

## Discussion

Despite the well-documented role of systemic LPS in driving pro-inflammatory or M1 macrophage polarization and subsequent dysfunction across numerous tissues, no studies to date have examined whether this same pathway contributes to dysregulation within the peripheral nervous system. Specifically, the literature is devoid of investigations examining whether chronic LPS exposure, secondary to gut dysbiosis and low-grade endotoxemia, induces M1-like macrophage polarization within peripheral nerve tissue. This represents a critical knowledge gap, particularly given the biological plausibility and clinical significance of such a mechanism.

The present review begins to address this absence by synthesizing mechanistic data on LPS-induced macrophage activation across diverse tissues and proposes a novel pathophysiologic axis linking gut dysbiosis to peripheral nerve injury, independent of the original cause of gut microbial imbalance.

Findings from this review demonstrate that chronic systemic LPS exposure may drive M1-like macrophage polarization, via conserved TLR4/NF-κB signaling pathways, across tissue types, leading to sustained tissue dysfunction – a pathologic process which is highly conserved throughout the body. Importantly, this classical-type macrophage activation persists even in immune-modulated environments such as pulmonary, adipose, and CNS tissues. In particular, LPS-induced neuroinflammation within the CNS has been shown to compromise the BBB and trigger glial activation. Prolonged activation of CNS microglia is well established as a source of neurotoxicity [19, 60]. In fact, chronic inflammation and CNS immune activation is a shared pathogenic route for multiple neurodegenerative diseases, including Alzheimer's disease, Parkinson's disease, Huntington's disease, and amyotrophic lateral sclerosis [61]. These findings collectively suggest that LPS-mediated disruption of the BNB, and associated peripheral neuropathy, is not only plausible but may be under-recognized.

Remarkably, animal models have shown that even a transient rise in circulating LPS, on the scale of hours, can initiate persistent neuroinflammatory cascades lasting *months* and culminating in progressive neuronal damage [14]. Given the structural and functional parallels between the BBB and BNB, including their shared reliance on tight junction proteins and glial modulation, similar self-sustaining inflammatory loops may exist within peripheral nerve tissue as well.

The incidence of peripheral neuropathy is steadily increasing, driven by an aging population, rising prevalence of chronic metabolic disorders, neurotoxic chemotherapeutic regimens, and emerging autoimmune syndromes [62]. Given that neuropathy is often progressive, debilitating, and resistant to conventional therapies [63], identifying novel and tractable pathophysiologic mechanisms is increasingly important. If chronic endotoxemia proves to be a causative or amplifying factor in peripheral nerve injury, then microbiome-modulating interventions – which range from targeted pre- or probiotics to dietary modulation and microbial metabolite therapeutics – could unlock unprecedented therapeutic potential. These strategies would not only represent a novel treatment, but would also be a paradigm shift, addressing neuropathy upstream via immunometabolic control.

This review has several limitations. First, the heterogeneity of included studies – spanning diverse disease models, tissues examined, LPS exposure (endogenous vs. exogenous LPS), and outcome measures – precluded formal quantitative meta-analysis. While our evidence-mapping approach enabled structured synthesis of mechanistic conservation, it cannot provide pooled effect estimates or establish the magnitude of any single pathway component. Second, many in-vivo studies relied on exogenous LPS administration (predominantly intraperitoneal injection), which bypasses the gut-barrier axis by design. Although this strengthens inference regarding downstream inflammatory and immune mechanisms, it limits direct generalizability to naturally occurring metabolic endotoxemia arising from gut dysbiosis. Thirdly, publication bias cannot be excluded; studies demonstrating null or negative findings may be underrepresented in the literature, potentially inflating perceived mechanistic concordance. Fourthly, more recent biological evidence suggests that macrophage polarization may not be phenotypically represented by binary M1/M2 states, but is better characterized as a more nuanced continuum. Therefore, the classification of traditionally M1, pro-inflammatory phenotyping in this paper may perhaps underestimate the full scope of LPS-mediated inflammatory response. However, as many papers included in this study were published prior to the broad acceptance of more nuanced macrophage activation, the authors felt it was more appropriate to describe all findings as “M1-like” or “pro-inflammatory” for descriptive consistency among historic and contemporary data. Finally, the search strategy may have excluded studies describing LPS-mediated inflammation using alternative terminology or outcome measures. However, the objective of this review was not to comprehensively catalog all manifestations of LPS-induced tissue injury, but rather to evaluate conservation of a specific mechanistic pathway linking LPS exposure to pro-inflammatory, M1-like macrophage activation. Consequently, pathway-associated markers such as TLR4, NF-κB, MyD88, CD86, and iNOS were intentionally incorporated into the search strategy to enrich for studies capable of informing mechanistic synthesis. While this approach may have reduced sensitivity, it increased specificity for the current review objective.

The consistency of LPS-induced M1-like macrophage polarization and downstream tissue dysfunction across diverse organ systems provides a compelling biological foundation for considering gut dysbiosis-driven endotoxemia as a potential upstream contributor to peripheral neuropathy. Although direct evidence within peripheral nerve tissue is currently lacking, the convergence of conserved immunologic pathways, barrier vulnerability, and clinical epidemiology suggests that this axis may warrant further experimental and translational investigation. Should the gut-immune-nerve axis exhibit pathway conservation, those findings could enable earlier risk stratification, biomarker development, or microbiome-targeted strategies aimed at preserving peripheral nerve integrity, particularly in populations predisposed to chronic dysbiosis or systemic inflammation.

## Data Availability

No datasets were generated or analysed during the current study.
